# ROS Modulating Inorganic Nanoparticles: A Novel Cancer Therapeutic Tool

**DOI:** 10.2174/2667387816666220506203123

**Published:** 2022-10-19

**Authors:** Maria John Newton Amaldoss, Charles Christopher Sorrell

**Affiliations:** 1 Prince of Wales Clinical School, UNSW Sydney, Sydney, NSW 2052, Australia;; 2 Adult Cancer Program, Lowy Cancer Research Centre, UNSW Sydney, Sydney, NSW 2052, Australia;; 3 Australian Centre for Nanomedicine, UNSW Sydney, Sydney, NSW 2052, Australia;; 4 School of Materials Science and Engineering, UNSW Sydney, Sydney, NSW 2052, Australia

**Keywords:** ROS, inorganic nanoparticles, therapeutic tool, chemodynamic therapy, tumor, cytotoxicity

## Abstract

The term “reactive oxygen species” (ROS) refers to a family of extremely reactive molecules. They are crucial as secondary messengers in both physiological functioning and the development of cancer. Tumors have developed the ability to survive at elevated ROS levels with significantly higher H_2_O_2_ levels than normal tissues. Chemodynamic therapy is a novel approach to cancer treatment that generates highly toxic hydroxyl radicals *via* a Fenton/Fenton-like reaction between metals and peroxides. Inorganic nanoparticles cause cytotoxicity by releasing ROS. Inorganic nanoparticles can alter redox homoeostasis by generating ROS or diminishing scavenging mechanisms. Internalized nanoparticles generate ROS in biological systems independent of the route of internalisation. This method of producing ROS could be employed to kill cancer cells as a therapeutic strategy. ROS also play a role in regulating the development of normal stem cells, as excessive ROS disturb the stem cells' regular biological cycles. ROS treatment has a significant effect on normal cellular function. Mitochondrial ROS are at the centre of metabolic changes and control a variety of other cellular processes, which can lead to medication resistance in cancer patients. As a result, utilising ROS in therapeutic applications can be a double-edged sword that requires better understanding.

## 1 ROLE OF ROS IN CANCER

Cancer treatment procedures have remained largely unchanged for a considerable length of time, relying primarily on radiation therapy and chemotherapy to date. However, significant efforts have been invested in the development of novel cancer treatments, which have improved therapeutic strategies through advanced drug delivery methods, gene silencing by siRNA delivery, and targeting new molecular targets and specific cellular organelles, such as mitochondria. One innovative therapeutic method is the use of reactive oxygen species (ROS), which are a class of molecules that exhibit reactivities greater than molecular oxygen. These species include, *inter alia*, the nonradicals hydrogen peroxide (H_2_O_2_) and hypochlorous acid (HOCl), and ozone (O_3_) as well as the free-radical-containing superoxide (^•^O_2_^−^) and hydroxyl (^•^OH) [1]. These species also play critical roles as secondary messengers in physiological functions, including the development of cancer.

Numerous studies have demonstrated that the concentrations of antioxidant enzymes are increased in cancer, indicating the relevance of oxidative stress in cancer [2]. In order to combat this oxidative stress, antioxidant enzymes are upregulated in the cancer microenvironment. Excessive levels of ROS can be controlled in both the tumor and physiological environment using antioxidant enzymes, such as superoxide dismutase (SOD), catalase (CAT), glutathione (GSH), and peroxiredoxins (Prxs), as well as nonenzymatic antioxidants, such as vitamins E and A [3, 4]. Additionally, enzymes, such as thioredoxin-1 (Trx-1) and GSH, are capable of restoring the normal function of redox-sensitive proteins by lowering the cysteine residues within those proteins [5]. SOD catalyses the conversion of ^•^O_2_^−^ to O_2_ and H_2_O_2_, whereas CAT catalyzes the subsequent conversion of H_2_O_2_ to H_2_O. The role of ROS as a tumor-promoting and tumor-suppressing agent is well established [6]. As some chemotherapeutic medicines used in cancer treatment involve an undetected ROS mechanism for apoptosis [7], this suggests that ROS may be used as tumor suppressants or novel therapeutic tools in cancer therapy. Alternatively, other studies have shown that higher ROS levels in cancer cells over an extended time aid in the development of resistance to cancer treatment [8]. Since ROS play a dual role as cancer suppressor and promoter, they can be viewed as a double-edged sword, where their prudent use or modulation in the cancer microenvironment can serve as an outstanding therapeutic tool, reshaping the landscape of cancer therapeutic strategies. Since the mitochondria, endoplasmic reticula (ER), peroxisomes, and NADPH oxidase (NOX) complexes on cell membranes are the cellular compartments most closely related to ROS formation [9], directing ROS**-**generating agents to these organelles can improve the therapeutic effect of ROS.

## 2 CANCER CHEMODYNAMIC THERAPY FOR ELEVATED ROS

ROS are present in the human body and have been implicated in ageing and a number of ailments, particularly neurological disorders [10, 11], cardiovascular diseases [12], and malignancies [13, 14]. Fenton and Fenton-like reactions involve the catalytic decomposition of H_2_O_2_ using the ferrous ion, resulting in the formation of additional ROS [15]. Chemodynamic therapy (CDT) is a novel approach to cancer treatment that utilizes a Fenton/Fenton-like reaction between metals and peroxides in order to generate highly toxic hydroxyl radicals (^•^OH) without the application of external energy, such as radiation [16, 17]. A metal-catalyzed free radical chain reaction is initiated by the by-products of aerobic respiration, such as H_2_O_2_, in this process. H_2_O_2_ can oxidize Fe^2+^ to form the hydroxide ion and highly reactive ^•^OH, which can kill cancer cells [1], although not at therapeutic concentrations. Consequently, an extrinsic metal ion supply may aid in achieving the desired therapeutic effect in CDT by creating sufficient concentrations of ^•^OH from H_2_O_2_. By inducing oxidative stress and generating oxidative damage to numerous biomolecules, including DNA and proteins, this highly oxidizing ^•^OH with strong oxidising can trigger cancer cell death. The Fenton/Fenton-like reactions can be represented by Equations 1 and 2 [18].


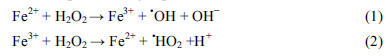


Tumors have developed the ability to survive at higher ROS levels because their H_2_O_2_ levels are substantially higher than those of normal tissue [19]. However, increasing the ROS levels in cancer cells to toxic levels causes oxidative stress-mediated cell death. As a result, the OH-based Fenton reaction can be considered to offer the best method to raise ROS levels to extremes for selective tumor therapy. Acid-sensitive Fe-based nanoparticles can deliver ferrous ions preferentially to tumor sites by taking advantage of tumors’ weak acidic environments, resulting in efficient and specific tumour treatment. The major ROS produced in the Fenton and Fenton-like reactions have been a longstanding subject of debate [15, 20-22]. These reactions can be complicated as they are influenced by a number of factors, including redox, ROS concentration, pH, ligand and buffer type, *etc*.

## 3 ROS-MEDIATED CANCER THERAPY WITH INORGANIC NANOPARTICLES

Inorganic nanoparticles, whether intentionally or unintentionally internalized by the body, cause cytotoxicity by creating ROS. Nanoparticles can change redox homoeostasis by generating ROS or decreasing ROS-scavenging mechanisms, regardless of the route of internalization, whether oral, parenteral, inhalation, or epidermal [23]. This ROS generation approach can be used as a therapeutic strategy to kill cancer cells. Maksoudian *et al.* [24] defined ROS oxidative stress as a three-step process, with the first stage involving a defensive reaction by detoxifying enzymes, such as SOD and CAT, to restore normal ROS homoeostasis. The second stage occurs when ROS levels exceed those of antioxidant defence systems, resulting in the production of proinflammatory cytokines and chemokines, which activate inflammatory cells, such as macrophages and neutrophils. The third stage is characterised by the formation of transient pores and a rise in intracellular Ca influx.

At the onset of ROS formation, the cytoplasmic Ca level is elevated, signalling the mitochondrial electron transport chain (ETC) to produce adenosine triphosphate (ATP) and ROS as by-products [9]. Disruption of ROS homoeostasis results in oxidative damage to a variety of cellular organelles and macromolecules, thus impairing normal cellular function. Excess ROS generation results in lipid peroxidation, protein oxidation, and nucleic acid oxidation, all of which damage the cell membrane, impair enzymatic activity, and hinder membrane permeability; it also enhances genotoxicity, which causes cell damage or death *via* autophagy, necrosis, or apoptosis.

When nanoparticles are delivered, they are internalized through a variety of physiological processes, including macropinocytosis, caveolin-clathrin independent pathway, caveolin-dependent pathway, clathrin-dependent pathway, and phagocytosis [25]. Internalized nanoparticles form early endosomes and then late endosomes, ultimately ending in endosomal escape. These processes allow inorganic nanoparticles to interact with cellular molecules, where the acidic environment and cellular biomolecules in cancer cells aid in the release of metal ions into the cellular environment, causing an imbalance in the local metal ion homoeostasis, gradually increasing the ROS levels. CuO, ZnO, and AgNO_3_ inorganic nanoparticles derived from transition metals have been shown to react with H_2_O_2_ and form ^•^OH *via* Fenton and Fenton-like reactions. These properties enable inorganic nanoparticles to be used in unique cancer therapy approaches. It has been reported widely that the redox properties of CeO_2_, which readily switches oxidation states between Ce^3+^ and Ce^4+^, are critical to the therapeutic capabilities of CeNPs [26, 27]. The antioxidant and prooxidant characteristics of CeNPs switch with pH between Ce^3+^ in the physiological environment to Ce^4+^ in the tumor microenvironment (TME) [28]. Ce is one of only three rare earth metals to exist in both 3+ (reduced) and 4+ (oxidized) valence states [29]. This redox switch enables it to exhibit antioxidant properties at a physiological pH value of ~7.4 while exhibiting prooxidant properties at the TME of pH value ~6.4 [26].

This feature of CeO_2_ nanoparticles is used in tumors for both cytoprotection of healthy cells and selective killing of cancer cells [30, 31]. Alternatively, CeO_2_ nanoparticles offer the potential to prevent cancer and other disorders by acting as a free radical scavenger and by reducing ROS levels, thus enabling their use as therapeutic agents [32, 33]. Additionally, it is possible to engineer inorganic nanoparticles in order to increase their solubility in biological environments by decreasing their stability through defect formation, size reduction, and other strategies. This approach also has the potential to increase the catalytic activity simultaneously. Success in this approach would enhance biodistribution and mitigate clearance difficulties. Simultaneously, fine-tuning the dissolution kinetics of inorganic nanoparticles can contribute to elevating the already-raised basal ROS levels in cancer cells, driving them above the lethal threshold. However, normal cells with normal basal ROS levels effectively counteract the increase in ROS levels through the action of antioxidant defence systems and detoxifying enzymes. As a result, normal cells sustain only transitory harm. Endogenous sources of ROS include mitochondrial respiration, inflammatory responses, microsomes, and peroxisomes. Free radicals are produced as a result of mitochondrial respiration, more significantly, Fenton and Fenton-like reactions mediated by transition metal ions [34]. A Fenton-like reaction can have a dose-dependent effect on numerous signal transduction pathways. While low or moderate ROS levels promote mitogenic signalling through reversible oxidation, excessive ROS levels result in the oxidation and peroxidation of nucleic acids and lipids, ultimately resulting in necrosis and cellular death.

### 3.1 ROS Mediated Photodynamic and Sonodynamic Therapy with Inorganic Nanoparticles

The mechanism of photodynamic therapy (PDT) involves the activation of a photosensitizer by an external source [35, 36]. The activation promotes the ground state of the photosensitizer molecule (PS) to the first excited singlet state (^1^PS^•^), which leads to a series of reactions that involve the intersystem crossing from the ^1^PS^•^ state to the triplet state (^3^PS^•^). This triplet state is relatively long-lived and critical to cell death *via* two pathways. In the Type I photochemical reactions, the transfer of electrons causes radicals to be formed, and these react with O_2_ to form ROS. In Type II photochemical reactions, the transfer of energy to O_2_ causes the highly reactive singlet oxygen (^1^O_2_) to form.

Sonodynamic therapy (SDT) operates in an analogous manner, except that a sonosensitizer replaces the photosensitizer, although most photosensitizers also are sonosensitizers. The complete mechanism of the SDT is unknown, but the current understanding is consistent with an underlying mechanism based on acoustic cavitation in an aqueous environment. Acoustic cavitation initiates the production of ROS to induce apoptosis. Recent work has demonstrated that a metal-organic-framework (MOF) double-layer hollow manganese silicate nanoparticle (DHMS) with high ROS yield, when activated with ultrasound, could be used as a theranostic device to provide multimodal imaging during sonodynamic therapy (SDT) [37]. The Mn in DHMS was oxidized by holes generated during an ultrasound, thereby augmenting ROS generation. Furthermore, there was a nearly 3.4-fold increase in the amount of ^1^O_2_, significantly raised ^•^OH levels, and generation of O_2_ in the TME. The latter both acted as contrast agents and served as a source of ROS.

Other work has reported that the combination of CDT and SDT to generate ROS can overcome the resistance to treatment commonly caused by the effect of a hypoxic TME [38, 39]. Thus, developing a therapeutic strategy that can alleviate tumour hypoxia is regarded as a critical strategy. For example, a novel nanozyme (MnO_2_ TPP-PEG) is capable of catalysing the reaction of H_2_O_2_ to O_2_ *via* a Fenton-like reaction, resulting in enhanced PDT upon light exposure [40]. Furthermore, catalysis of H_2_O_2_ at the tumor site can generate cytotoxic ^•^OH, resulting in effective CDT to augment the ROS-mediated anticancer effect.

### 3.2 Modification of Tumor Microenvironment Bb Redox-Active Nanoparticles

Although current research has focused on neoplastic transformation and tumour growth, an unknown factor is the role of contact between cancer cells and the stromal microenvironment in tumour growth. While it is well established that ROS play critical roles in all stages of cancer development, until recently, the molecular mechanisms underpinning the ROS-dependent tumor/stroma interaction during tumour progression and its potential therapeutic regulation to inhibit tumor invasion are not well understood. TGFβ1 enhances intracellular ^•^O_2_ concentration by activating NAD(P)H oxidase in human lung and skin fibroblasts, resulting in the development of myofibroblasts. For skin-derived tumor cells, the effect of dextran-coated CeO_2_ nanoparticles in the context of preventing myofibroblast development and tumor invasion associated with the tumor/stroma interaction was studied [41]. This study revealed that CeO_2_ nanoparticles play a dual role based on the Warburg effect, benefiting stromal cells (fibroblasts) while being cytotoxic to tumor cells.

Angiogenesis is a critical process involved in a variety of physiological and pathological processes. Pathological conditions, such as psoriasis, diabetic retinopathy, and cancer, exhibit unregulated angiogenesis, which is critical in cancer because it supplies nourishment and removes metabolic waste from the tumor site. Vascular endothelial growth factor (VEGF) is one of the most important proangiogenic factors, and it acts as a mitogen for vascular endothelial cells *in vitro* and as an angiogenic factor *in vivo* [42]. Nanoceria has been reported to exhibit anticancer and anti-invasive effects [43]. Recently, it has been proposed that ROS control VEGF production and thus regulate tumor-induced angiogenesis. Nanoceria has been tested *in vitro* and *in vivo* against ovarian cancer, where it was observed to inhibit growth-factor-mediated migration and invasion of SKOV3 cells, VEGF-induced proliferation, and capillary tube formation cells [44]. Most importantly, nanoceria inhibited tumor growth *in vivo* by inhibiting angiogenesis, specifically by targeting vascular endothelial cells.

### 3.3 Antioxidant Inorganic Nanoparticle for Reducing UV Radiation Toxicity

Exposure to UV radiation causes various skin responses, including premature skin aging and carcinogenesis, both of which are driven by elevated ROS. Nanomaterials have been tested for the prevention of photoaging, where these nanozymes act as antioxidants that scavenge excess ROS produced in the skin. Low-dose nanoceria has been tested for its protection profile against oxidative redox imbalance caused by exposure to UV-A (UVA), revealing protective effects on cell survival, migration, and proliferation [45]. Pretreatment of fibroblast cells (L929) with nanoceria exposed to UV-A demonstrated negligible cytotoxicity while protecting against cell death. Nanoceria also inhibited ROS production immediately after irradiation and restored SOD activity and GSH level for up to 48 h. Similarly, engineered CeO_2_ nanoparticles for radiation protection, specifically by modulating neutrophil oxidative response under low-dose UV-B, were investigated [46]. These data revealed that even low doses of UV-B activate the neutrophil oxidative response and that the antioxidant activities of engineered CeO_2_ nanoparticles can inhibit the effects of NADPH oxidase activation while maintaining SOD and CAT mimetic activity [47]. These antioxidant therapies have also been observed to enhance the neuroprotection by nanoceria in *in vivo* animal models of Alzheimer’s disease [48].

## 4 CHALLENGES AND FUTURE DIRECTIONS

Another significant problem is the use of dependable and effective ROS-detection systems [49]. For the detection or estimation of ROS formation, several direct and indirect approaches have been developed [50]. Unfortunately, the levels of ROS involved in oxidative stress activity are so low that accurate detection or quantification of these short-lived species is extremely difficult [51]. In general, spectrophotometry and fluorometry generally have been used. These approaches are based on the change in chromogen absorption owing to ROS interaction and probe characteristics. Numerous intracellular ROS indicators, ranging from secondary assays to primary observable indicators based on real-time fluorescence, are available [52]. In order to create an effective ROS-detection technology, it is necessary to harmonize the existing ROS detection technologies so that there is a common baseline for assessment of biological systems and accurate measurement of ROS changes, regardless of the method used [1]. The development of robust and rapid methodologies will aid in the reduction of technical errors associated with challenging measurement and quantification of ROS, particularly as ROS levels can change spontaneously in biological environments. The combination of optimized ROS-modifying nanoparticles and validated ROS-detection systems is expected to increase the therapeutic efficacy of ROS-based therapies.

Although inorganic nanoparticle-mediated ROS cancer treatment is a promising technique, clinical implementation remains hindered. The prooxidant therapy mediated by inorganic nanoparticles is sufficient to disrupt ROS homoeostasis and drive cancer cells to oxidative stress-induced cell death [53]. However, it is critical to understand the effects on normal cells. As stem cells are vulnerable to ROS, targeting specific ROS pathways rather than non-targeted ROS therapy may be a superior approach. ROS are also important in determining how normal stem cells will develop, where stem cells require a relatively low amount of ROS to maintain quiescence and self-renewal. Increased levels of ROS promote stem cell growth, differentiation, senescence, apoptosis, and cell debris removal in dose-dependent ways [54]. Consequently, the ROS levels are regulated strictly by cells in order to preserve homoeostasis, which aids in the regulation of tissue repair and thus the organism's life span. Thus, ROS-based therapies aimed at specific antioxidant pathways or molecular targets are likely to point to the future of improved therapeutic outcomes.

## CONCLUSION

ROS have well-established roles as tumor-promoting and tumor-suppressing agents. Catalytic agents, both biological and synthetic, can be employed to regulate excess ROS in both the physiological environment and the TME. As inorganic nanoparticles cause cytotoxicity by releasing ROS, this method of ROS generation can be employed to kill cancer cells as a therapeutic treatment. Nanoparticles can be engineered to increase both their solubility in biological environments and their catalytic activity. Despite the advantages of inorganic-nanoparticle-mediated ROS cancer therapies, their translation to clinical applications remains yet to be realized. However, they offer a range of alternative approaches to therapy, including the leveraging of specific antioxidant pathways and molecular targeting in ROS therapies.

## LIST OF ABBREVIATIONS

ATP: Adenosine triphosphate
CAT: Catalase
CDT: Chemodynamic therapy
ER: endoplasmic reticula
ETC: Electron transport chain
HOCl: Hypochlorous acid
MOF: Metal-organic-framework
NOX: NADPH oxidase
PDT: Photodynamic therapy
ROS: Reactive oxygen species
SOD: Sonodynamic therapy
SOD: Superoxide dismutase
TME: Tumor microenvironment
VEGF: Vascular endothelial growth factor

## References

[1] Chio I.I.C., Tuveson D.A. (2017). ROS in Cancer: The Burning Question.. Trends Mol. Med..

[2] Islam M.O., Bacchetti T., Ferretti G. (2019). Alterations of Antioxidant Enzymes and Biomarkers of Nitro-oxidative Stress in Tissues of Bladder Cancer.. Oxid. Med. Cell. Longev..

[3] He L., He T., Farrar S., Ji L., Liu T., Ma X. (2017). Antioxidants Maintain Cellular Redox Homeostasis by Elimination of Reactive Oxygen Species.. Cell. Physiol. Biochem..

[4] Karihtala P., Soini Y. (2007). Reactive oxygen species and antioxidant mechanisms in human tissues and their relation to malignancies.. APMIS.

[5] Castaldo S.A., Freitas J.R., Conchinha N.V., Madureira P.A., The Tumorigenic Roles of the Cellular REDOX Regulatory Systems (2016). Oxid. Med. Cell. Longev..

[6] Wang J., Yi J. (2008). Cancer cell killing via ROS: To increase or decrease, that is the question.. Cancer Biol. Ther..

[7] Conklin K.A. (2004). Chemotherapy-Associated Oxidative Stress: Impact on Chemotherapeutic Effectiveness.. Integr. Cancer Ther..

[8] de Sá Junior P.L., Câmara D.A.D., Porcacchia A.S., Fonseca P.M.M., Jorge S.D., Araldi R.P., Ferreira A.K. (2017). The Roles of ROS in Cancer Heterogeneity and Therapy.. Oxid. Med. Cell. Longev..

[9] Zorov D.B., Juhaszova M., Sollott S.J. (2014). Mitochondrial reactive oxygen species (ROS) and ROS-induced ROS release.. Physiol. Rev..

[10] Kepp K.P. (2012). Bioinorganic Chemistry of Alzheimer’s Disease.. Chem. Rev..

[11] Savelieff M.G., Nam G., Kang J., Lee H.J., Lee M., Lim M.H. (2019). Development of Multifunctional Molecules as Potential Therapeutic Candidates for Alzheimer’s Disease, Parkinson’s Disease, and Amyotrophic Lateral Sclerosis in the Last Decade.. Chem. Rev..

[12] Sawicki K.T., Chang H.C., Ardehali H., Role of Heme in Cardiovascular Physiology and Disease J. Am. Heart Assoc..

[13] Torti S.V., Torti F.M. (2013). Iron and cancer: more ore to be mined.. Nat. Rev. Cancer.

[14] Sun H., Zhang C., Cao S., Sheng T., Dong N., Xu Y. (2018). Fenton reactions drive nucleotide and ATP syntheses in cancer.. J. Mol. Cell Biol..

[15] Chen H-Y. (2019). Why the Reactive Oxygen Species of the Fenton Reaction Switches from Oxoiron(IV) Species to Hydroxyl Radical in Phosphate Buffer Solutions? A Computational Rationale.. ACS Omega.

[16] Xie A., Li H., Hao Y., Zhang Y. (2021). Tuning the Toxicity of Reactive Oxygen Species into Advanced Tumor Therapy.. Nanoscale Res. Lett..

[17] Tian Q., Xue F., Wang Y., Cheng Y., An L., Yang S., Chen X., Huang G. (2021). Recent advances in enhanced chemodynamic therapy strategies.. Nano Today.

[18] Enami S., Sakamoto Y., Colussi Agustín J. (2014). Fenton chemistry at aqueous interfaces.. Proc. Natl. Acad. Sci. USA.

[19] Aykin-Burns N., Ahmad I.M., Zhu Y., Oberley L.W., Spitz D.R. (2009). Increased levels of superoxide and H2O2 mediate the differential susceptibility of cancer cells versus normal cells to glucose deprivation.. Biochem. J..

[20] Haber F., Weiss J., Pope W.J. (1934). The catalytic decomposition of hydrogen peroxide by iron salts.. Proceedings of the Royal Society of London. Series A - Mathematical and Physical Sciences.

[21] Walling C. (1998). Intermediates in the Reactions of Fenton Type Reagents.. Acc. Chem. Res..

[22] Sawyer D.T., Sobkowiak A., Matsushita T. (1996). Metal [MLx; M = Fe, Cu, Co, Mn]/Hydroperoxide-Induced Activation of Dioxygen for the Oxygenation of Hydrocarbons: Oxygenated Fenton Chemistry.. Acc. Chem. Res..

[23] Tee J.K., Ong C.N., Bay B.H., Ho H.K., Leong D.T. (2016). Oxidative stress by inorganic nanoparticles.. WIREs Nanomedicine and Nanobiotechnolog.

[24] Maksoudian C., Saffarzadeh N., Hesemans E., Dekoning N., Buttiens K., Soenen S.J. (2020). Role of inorganic nanoparticle degradation in cancer therapy.. Nanoscale Adv..

[25] Foroozandeh P., Aziz A.A. (2018). Insight into Cellular Uptake and Intracellular Trafficking of Nanoparticles.. Nanoscale Res. Lett..

[26] Mehmood R., Ariotti N., Yang J.L., Koshy P., Sorrell C.C. (2018). pH-Responsive Morphology-Controlled Redox Behavior and Cellular Uptake of Nanoceria in Fibrosarcoma.. ACS Biomater. Sci. Eng..

[27] Xu C., Qu X. (2014). Cerium oxide nanoparticle: a remarkably versatile rare earth nanomaterial for biological applications.. NPG Asia Mater..

[28] Sadidi H., Hooshmand S., Ahmadabadi A., Javad Hosseini S., Baino F., Vatanpour M., Kargozar S. (2020). Cerium Oxide Nanoparticles (Nanoceria): Hopes in Soft Tissue Engineering.. Molecules.

[29] Tsunekawa S., Sivamohan R., Ohsuna T., Takahashi H., Tohji K. (1999). In Materials Science Forum.. Trans Tech Publ.

[30] Gao Y., Chen K., Ma J-L., Gao F. (2014). Cerium oxide nanoparticles in cancer.. OncoTargets Ther..

[31] Thakur N., Manna P., Das J. (2019). Synthesis and biomedical applications of nanoceria, a redox active nanoparticle.. J. Nanobiotechnology.

[32] Nelson B.C., Johnson M.E., Walker M.L., Riley K.R., Sims C.M. (2016). Antioxidant Cerium Oxide Nanoparticles in Biology and Medicine.. Antioxidants (Basel).

[33] Kabir M.S., Munroe P., Gonçales V., Zhou Z., Xie Z. (2018). Structure and properties of hydrophobic CeO2−x coatings synthesized by reactive magnetron sputtering for biomedical applications.. Surf. Coat. Tech..

[34] Canaparo R., Foglietta F., Limongi T., Serpe L., Biomedical Applications of Reactive Oxygen Species Generation by Metal Nanoparticles (2020). Materials (Basel, Switzerland).

[35] Liu J., Du P., Liu T., Córdova Wong B.J., Wang W., Ju H., Lei J. (2019). A black phosphorus/manganese dioxide nanoplatform: Oxygen self-supply monitoring, photodynamic therapy enhancement and feedback.. Biomaterials.

[36] Plaetzer K., Krammer B., Berlanda J., Berr F., Kiesslich T. (2009). Photophysics and photochemistry of photodynamic therapy: fundamental aspects.. Lasers Med. Sci..

[37] Pan X., Wang W., Huang Z., Liu S., Guo J., Zhang F., Yuan H., Li X., Liu F., Liu H. (2020). MOF-Derived Double-Layer Hollow Nanoparticles with Oxygen Generation Ability for Multimodal Imaging-Guided Sonodynamic Therapy.. Angew. Chem. Int. Ed..

[38] Wang W., Lin L., Ma X., Wang B., Liu S., Yan X., Li S., Tian H., Yu X. (2018). Light-Induced Hypoxia-Triggered Living Nanocarriers for Synergistic Cancer Therapy.. ACS Appl. Mater. Interfaces.

[39] Cui Q., Wang J.Q., Assaraf Y.G., Ren L., Gupta P., Wei L., Ashby C.R., Yang D.H., Chen Z.S. (2018). Modulating ROS to overcome multidrug resistance in cancer.. Drug Resist. Updat..

[40] Chen Y., Cai Y., Yu X., Xiao H., He H., Xiao Z., Wang Y., Shuai X. (2021). A photo and tumor microenvironment activated nano-enzyme with enhanced ROS generation and hypoxia relief for efficient cancer therapy.. J. Mater. Chem. B Mater. Biol. Med..

[41] Alili L., Sack M., Karakoti A.S., Teuber S., Puschmann K., Hirst S.M., Reilly C.M., Zanger K., Stahl W., Das S., Seal S., Brenneisen P. (2011). Combined cytotoxic and anti-invasive properties of redox-active nanoparticles in tumor–stroma interactions.. Biomaterials.

[42] Alili L., Sack M., von Montfort C., Giri S., Das S., Carroll K.S., Zanger K., Seal S., Brenneisen P. (2012). Downregulation of Tumor Growth and Invasion by Redox-Active Nanoparticles.. Antioxid. Redox Signal..

[43] Sack M., Alili L., Karaman E., Das S., Gupta A., Seal S., Brenneisen P. (2014). Combination of Conventional Chemotherapeutics with Redox-Active Cerium Oxide Nanoparticles—A Novel Aspect in Cancer Therapy.. Mol. Cancer Ther..

[44] Giri S., Karakoti A., Graham R.P., Maguire J.L., Reilly C.M., Seal S., Rattan R., Shridhar V. (2013). Nanoceria: A Rare-Earth Nanoparticle as a Novel Anti-Angiogenic Therapeutic Agent in Ovarian Cancer.. PLoS One.

[45] Ribeiro F.M., de Oliveira M.M., Singh S., Sakthivel T.S., Neal C.J., Seal S., Ueda-Nakamura T., Lautenschlager S.O.S., Nakamura C.V. (2020). Ceria Nanoparticles Decrease UVA-Induced Fibroblast Death Through Cell Redox Regulation Leading to Cell Survival, Migration and Proliferation.. Front. Bioeng. Biotechnol..

[46] Peloi K.E., Contreras Lancheros C.A., Nakamura C.V., Singh S., Neal C., Sakthivel T.S., Seal S., Lautenschlager S.O.S. (2020). Antioxidative photochemoprotector effects of cerium oxide nanoparticles on UVB irradiated fibroblast cells.. Colloids Surf. B Biointerfaces.

[47] Peloi K.E., Ratti B.A., Nakamura C.V., Neal C.J., Sakthivel T.S., Singh S., Seal S., de Oliveira Silva Lautenschlager S. (2021). Engineered nanoceria modulate neutrophil oxidative response to low doses of UV-B radiation through the inhibition of reactive oxygen species production.. J. Biomed. Mater. Res. A.

[48] Dowding J.M., Song W., Bossy K., Karakoti A., Kumar A., Kim A., Bossy B., Seal S., Ellisman M.H., Perkins G., Self W.T., Bossy-Wetzel E. (2014). Cerium oxide nanoparticles protect against Aβ-induced mitochondrial fragmentation and neuronal cell death.. Cell Death Differ..

[49] Olowe R., Sandouka S., Saadi A., Shekh-Ahmad T. (2020). Approaches for Reactive Oxygen Species and Oxidative Stress Quantification in Epilepsy..

[50] Batandier C., Fontaine E., Kériel C., Leverve X.M. (2002). Determination of mitochondrial reactive oxygen species: methodological aspects.. J. Cell. Mol. Med..

[51] Valgimigli L., Pedulli G.F., Paolini M. (2001). Measurement of oxidative stress by EPR radical-probe technique.. Free Radic. Biol. Med..

[52] Yang H., Villani R.M., Wang H., Simpson M.J., Roberts M.S., Tang M., Liang X. (2018). The role of cellular reactive oxygen species in cancer chemotherapy.. J. Exp. Clin. Cancer Res..

[53] Reczek C.R., Chandel N.S. (2017). The Two Faces of Reactive Oxygen Species in Cancer.. Annu. Rev. Cancer Biol..

[54] Zhou D., Shao L., Spitz D.R. (2014). Reactive oxygen species in normal and tumor stem cells.. Adv. Cancer Res..

